# *Tripius gyraloura* n. sp. (Aphelenchoidea: Sphaerulariidae) parasitic in the gall midge *Lasioptera donacis* Coutin (Diptera: Cecidomyiidae)

**DOI:** 10.1007/s11230-014-9524-1

**Published:** 2014-10-10

**Authors:** George Poinar, Donald B. Thomas

**Affiliations:** 1 Department of Zoology, Oregon State University, Corvallis, OR 97331 USA; 2 USDA-ARS Cattle Fever Tick Research Laboratory, 22675 N. Moorefield Rd, Edinburg, TX 78596 USA

## Abstract

A new nematode, *Tripius gyraloura* n. sp., is described from the arundo gall midge, *Lasioptera donacis* Coutin (Diptera: Cecidomyiidae). This gall midge is being considered as a biological control agent for use in North America against the introduced giant reed *Arundo donax* (L.) (Poaceae: Cyperales). Thus the present study was initiated to investigate a nematode parasite that was unknown at the time studies with *L. donacis* were initiated. The new species has a rapid development in the fly host and the mature parasitic female nematodes evert their uterine cells in the hosts’ hemolymph. Because large numbers of nematodes sterilise the host, eradication of the parasite from laboratory colonies of the midge may be necessary before populations of the fly are released.

## Introduction

The Old World giant reed, *Arundo donax* (L.), native to the Mediterranean, was introduced into North America in the 16th Century (Tarin et al., 2013) and since then has become a noxious weed. Attempts have and are being made to find insects that feed on this invasive plant and may be useful in reducing its negative impacts (Seawright et al., 2009). The arundo gall midge, *Lasioptera donacis* Coutin (Diptera: Cecidomyiidae), is one of the insects under consideration. Studies were initiated at a quarantine facility in Texas to determine the potential of *L. donacis* imported from Europe as a biological control agent of giant reed. During these investigations, it was discovered that some of the larvae, pupae and adults of *Lasioptera donacis* sent from Europe were infected by a single species of parasitic nematode of the genus *Tripius* Chitwood, 1935 (Sphaerulariidae). The present work describes this nematode and discusses its biology in relation to the fly host.

## Materials and methods

Stems of giant reed infested by *Lasioptera donacis* were collected in France, Italy and Greece, and shipped to Texas under permit where they were maintained at the USDA quarantine facility at Moore Air Base, Edinburg, TX. Larvae and pupae of *Lasioptera donacis* found in the leaf sheath, or adults emerging from these stems, were killed and fixed in 5% formalin or 95% ethanol. These specimens were forwarded to the senior author who dissected them for various stages of the nematode. Measurements were made on fixed nematodes that were processed to glycerine by the evaporation method. The following description is based on infective stage females and parasitic females removed from host material. Examination and photographs were made with a Nikon stereoscopic microscope SMZ-10-R at magnification of ×80 and a Nikon Optiphot microscope at magnification of ×1,000. Helicon Focus was used to stack photos for better depth of field. All measurements are in micrometres unless otherwise specified and are presented as the range followed by the mean in parentheses.

- **Order Tylenchida Thorne, 1949**
- **Superfamily Sphaerularioidea Lubbock, 1861**
- **Family Sphaerulariidae Lubbock, 1865**

## *Tripius gyraloura* n. sp.

*Type-host*: Arundo gall midge, *Lasioptera donacis* Coutin (Diptera: Cecidomyiidae).

*Type-locality*: Peraia, Greece.

*Type-material*: Free-living female (USDANL # T-661t) and paratype (parasitic female USDANL # T-6122p) deposited in the USDA Nematode Laboratory, Beltsville, Maryland. Paratypes are deposited in the first author’s collection.

*Etymology*: The specific epithet is from the Greek “gyros” (round) and the Greek “oura” (tail) in reference to the rounded tail tip on the parasitic juveniles, males and free-living females.

### Description (Figs. 1–7)

*General*

**Figs. 1–2:**
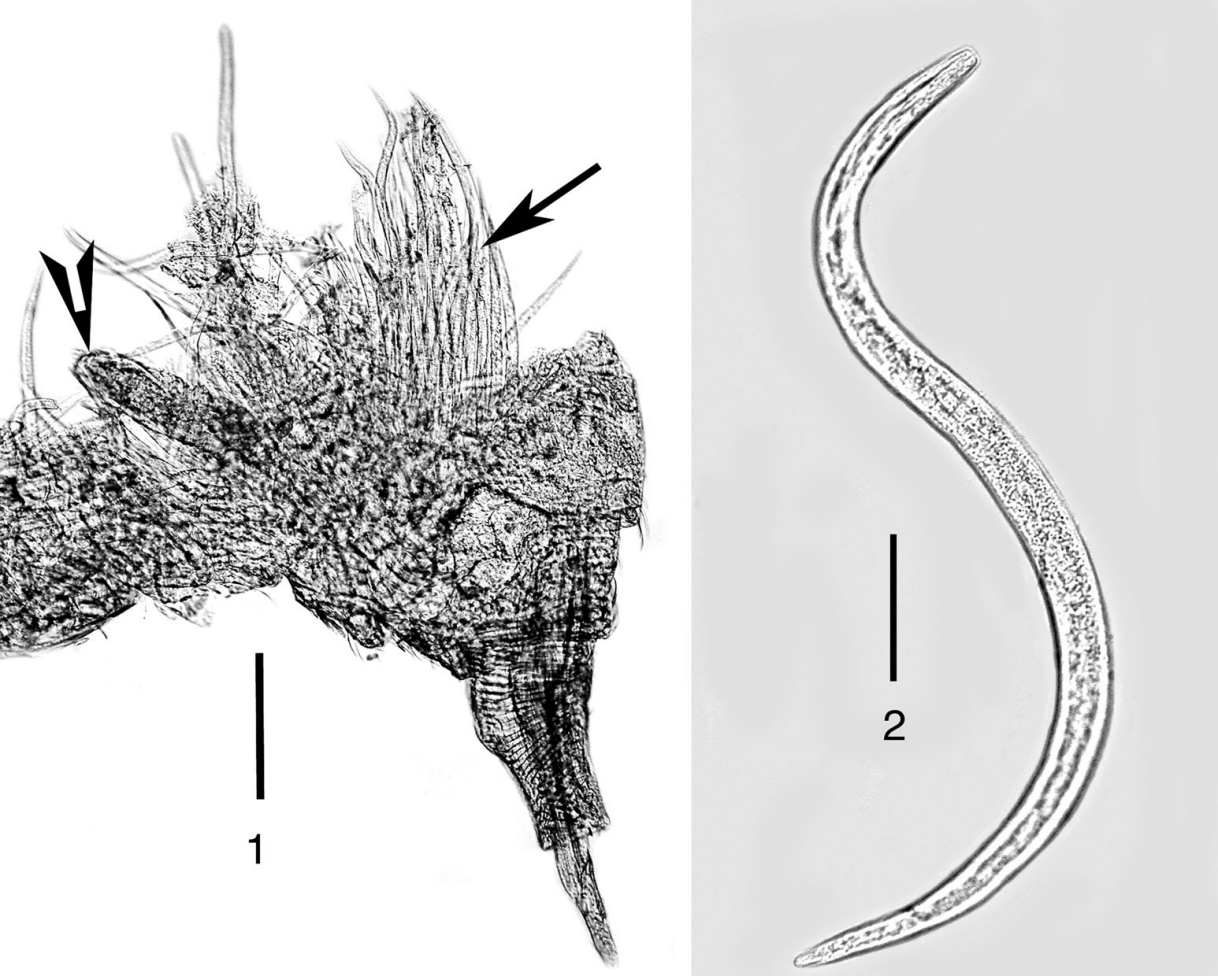
*Tripius gyraloura* n. sp. 1, Cluster of parasitic juveniles (arrow) in a dissected female *Lasioptera donacis* (arrowhead shows anterior end of a mature parasitic female); 2, Free-living infective stage female. *Scale-bars*: 1, 144 µm; 2, 39 µm

Small nematodes with fine longitudinal and transverse cuticular striations. Stylet present, slightly knobbed in female; two subventral pharyngeal glands; anus conspicuous; all stages with rounded tails. Reproductive tract in female divided into a terminal ovary followed by oviduct, seminal vesicle or spermatheca containing sperm, quadricolumella and finally uterus, vagina and vulva (Fig. 4). Oviparous, with homogonic life-cycle (parasitic cycle only; no alternating free-living or plant feeding life-cycle generations). Bisexual; parasitic females evert uterine cells into host haemocoel during development.

**Figs. 3–4:**
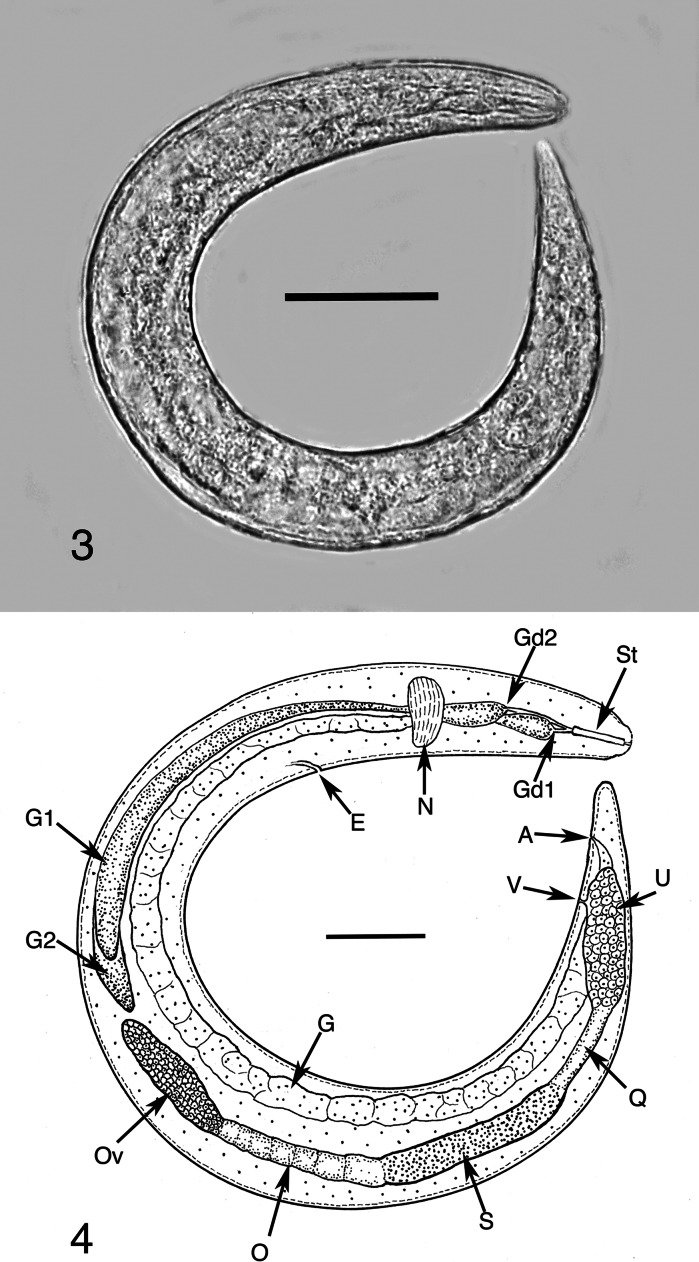
*Tripius gyraloura* n. sp. 3, Infective stage female shortly after entering the body cavity of a larva of *Lasioptera donacis*; 4, Drawing of the female shown in Fig. 3. *Abbreviations*: A, anus; E, excretory pore; G, gut; Gd1, anterior gland duct; Gd2, posterior gland duct; G1, anterior subventral gland; G2, posterior subventral gland; N, nerve-ring; O, oviduct; Ov, ovary; Q, quadricolumella; S, seminal receptacle (spermatheca); St, stylet; U, uterus; V, vulva. *Scale-bars*: 3, 41 µm; 4, 26 µm

*Free-living infective stage female* (n = 10). Stylet well developed, length 8–15 (10); pharyngeal glands prominent; first subventral gland duct positioned immediately posterior to stylet base; second subventral gland duct positioned about 1.5 stylet lengths posterior to stylet base. Body length 297–360 (325); greatest body width 16–26 (20); distance from anterior extremity to nerve-ring 36–67 (50); distance from anterior extremity to excretory pore 50–88 (66); length of first subventral gland 133–160 (145); length of second subventral gland 147–166 (155); tail length 20–34 (28); % vulva 74–88 (82)%.

*Free-living male* (n = 6). Stylet very faint, lacking basal thickenings; pharyngeal glands greatly reduced; testis outstretched, spicules paired, slightly curved, faintly cephalated; gubernaculum short, bursa absent. Body length 415–428 (420); body width 10–18 (14); stylet length 7–10 (8); distance from anterior extremity to nerve-ring, 28–38 (35); distance from anterior extremity to excretory pore, 38–47 (40); length of spicules 6–10 (8); length of gubernaculum 3–4 (3), length of tail 30–43 (37); tail tip rounded.

*Parasitic female* (n = 8). Long, swollen, oviparous nematodes with cephalic and caudal region often narrowed. Stylet well developed. Uterine cells partly expelled through vulva into host haemocoel. Body length 0.6–2.0 (0.9) mm, greatest width 48–135 (58); % vulva 85–96 (89)%; eggs ellipsoidal, length, 70–76 × 22–29 (73 × 25). Gut cell boundaries difficult to decipher; gut probably syncytial.

*Fourth stage parasitic juveniles* (n = 10). Body length 290–430 (360), width 10–26 (17); enclosed in fourth stage cuticle.

### Remarks

Previous authors have considered the portion of the uterus positioned posterior to the vulva as a post-uterine sac. Here it is actually part of the normal uterus that is extended posteriorly and not a specialised structure. The quadricolumella is that portion of the oviduct that secretes the egg shell. While there is an obvious anus, the gut is not connected to the mouth and appears to be a food storage organ, providing the body with nutrients absorbed from the host *via* the cuticle. The uterine cells are never completely expelled through the vulva as they are in *Tripius gibbosus* (Leuckart, 1887) (see Leuckart, 1887).

Currently there are two described species in the genus *Tripius*. *Tripius gyraloura* n. sp. differs from *T. gibbosus* in possessing a stylet with faint basal thickenings and a pair of subventral pharyngeal glands with duct openings located directly or slightly behind the stylet base. The stylet of the free-living infective stage female of *T. gibbosus* lacks basal thickenings and the single pharyngeal gland duct opening is positioned posterior to the excretory pore. In addition, all stages of *T. gyraloura* have a rounded tail tip, while those of *T. gibbosus* have a pointed tail tip. Also, the infective stage female of *T. gyraloura* has a much shorter tail (28 *vs* 75 µm in *T. gibbosus*) and a longer % vulva (mean 82 *vs* 70% in *T. gibbosus*) (see Leuckart, 1887).

The infective female of *Tripius sciarae* (Bovien, 1944) has a stylet with a tripartite base, whereas the stylet base of *T. gyraloura* n. sp. is slightly thickened but not tripartite. The ventral gland openings of *T. sciarae* are two stylet lengths posterior to the stylet base whereas in *T. gyraloura* they open immediately and 1.5 stylet lengths posterior to the stylet. In addition the ducts of the two subventral glands are separate in *T. gyraloura* but are opposite in *T. sciarae*. Free-living stages of *T. gyraloura* have a rounded tail tip, while those of *T. scariae* have a pointed tail tip. Also, the infective stage female of *T. gyraloura* has a much shorter tail, the proportion of tail length to body length is 1/11 while it is 1/5 in *T. sciarae* and the position of the vulva is 74–88% in *T. gyraloura* while this value is 65–72% in *T. sciarae* (see Bovien, 1944; Poinar, 1965). The latter species is a parasite of sciarid flies while *T. gibbosus* is a parasite of gall midges and has not been recovered since its discovery in 1887 (Poinar, 1975).

The only other nematode parasite infecting a member of the family Cecidomyiidae is the fossil Tertiary mermithid *Heydenius cecidomyae* Poinar, 2011 entombed in 40–50 million year-old amber from the Baltic Region (Poinar, 2011). Mermithid nematodes have no resemblance to the species of *Tripius* presented here.

### Life-cycle and effect on host

After fertilisation in the environment, the males die and the females utilise the secretions in their subventral pharyngeal glands to penetrate the body wall of host larvae. This method of entrance was shown to occur in the infective females of the sciarid parasite *T.* *sciarae* in England (Poinar & Doncaster, 1965). Most juvenile development occurs in the pupal and adult stages of the flies and infected adult hosts are often packed with spent females and fourth stage juveniles (Fig. 1). We suggest that the mature juveniles are initially expelled out of the host’s ovipositor but also exit the fly when it dies, as was noted with *T. scariae* (see Poinar & Doncaster, 1965). Many infected adult flies have their body cavities filled with nematodes and heavily parasitised female hosts show no or little sign of egg development. Thus the nematode can be considered an important natural enemy of *Lasioptera donacis*. Similar host effects were noted with *T. gibbosus* (see Leuckart, 1887) and *T. sciarae* (see Bovien, 1944; Poinar 1965).

## Discussion

The present study establishes a third species in the genus *Tripius*. Two of these (*T.* *gibbosus* and *Tripius gyraloura* n. sp.) infect flies of the family Cecidomyiidae while *T.* *sciarae* is restricted to sciarid flies. While the latter species is fairly common, *T. gibbosus* is not known except from its original description by Leuckart in 1887.

There is presumably a complex of closely related *Tripius* species and strains that parasitise cecidomyiids globally even though *Tripius gyraloura* n. sp. is only the second species reported from gall midges. While in the same family (Cecidomyiidae) and subfamily (Cecidomyiinae), the two gall midge hosts of *T. gyraloura* n. sp. and *T. gibbosus*, namely *Lasioptera donacis* Coutin and *Cecidomyia pini* DeGeer respectively, are only distantly related. *Lasioptera* is in the supertribe Lasiopteridi while *Cecidomyia* is in the supertribe Cecidomyiidi.

**Figs. 5–6:**
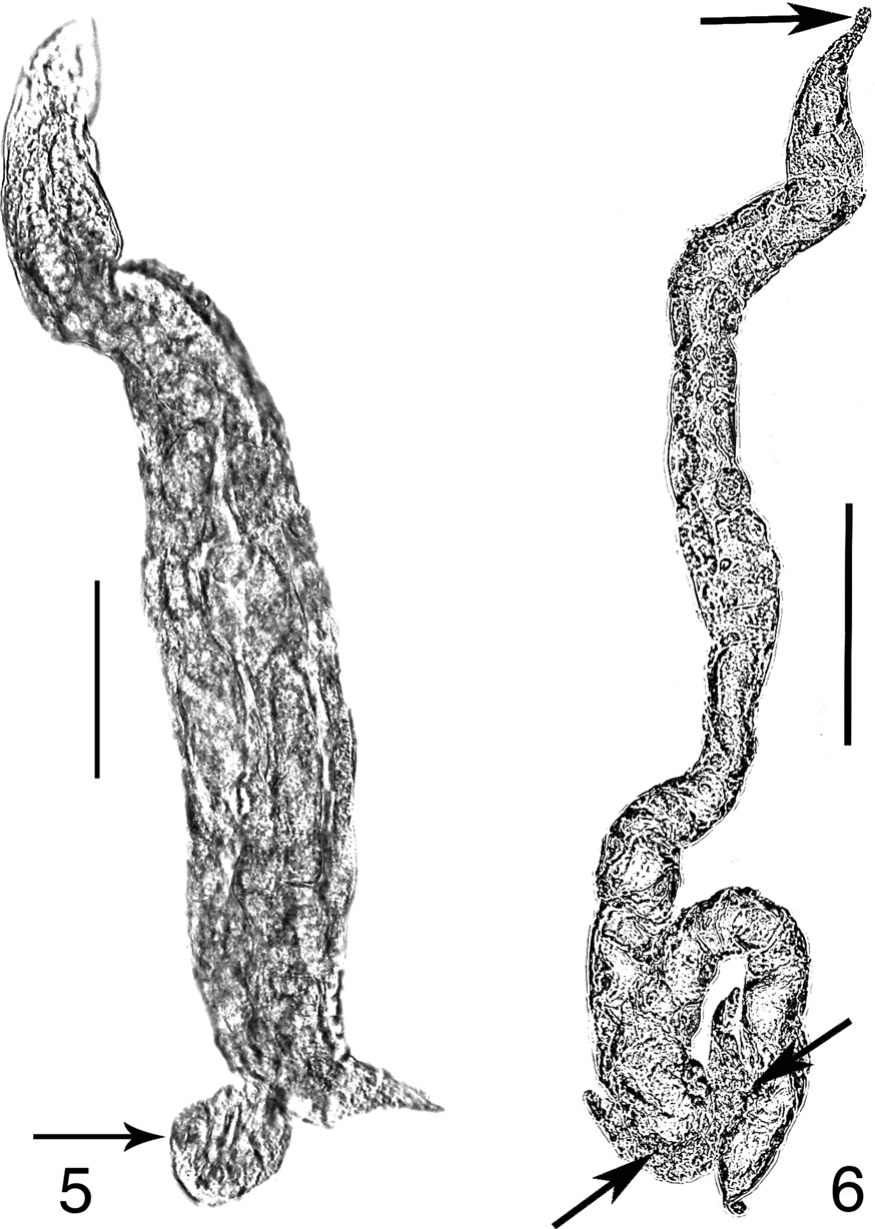
*Tripius gyraloura* n. sp. 5, Mature parasitic female with protruding uterine cells (arrow); 6, Elongate mature parasitic female (top arrow shows head; bottom left arrow shows protruded uterus; bottom right arrow shows vulva). *Scale-bars*: 5, 247 µm; 6, 35 µm

**Fig. 7:**
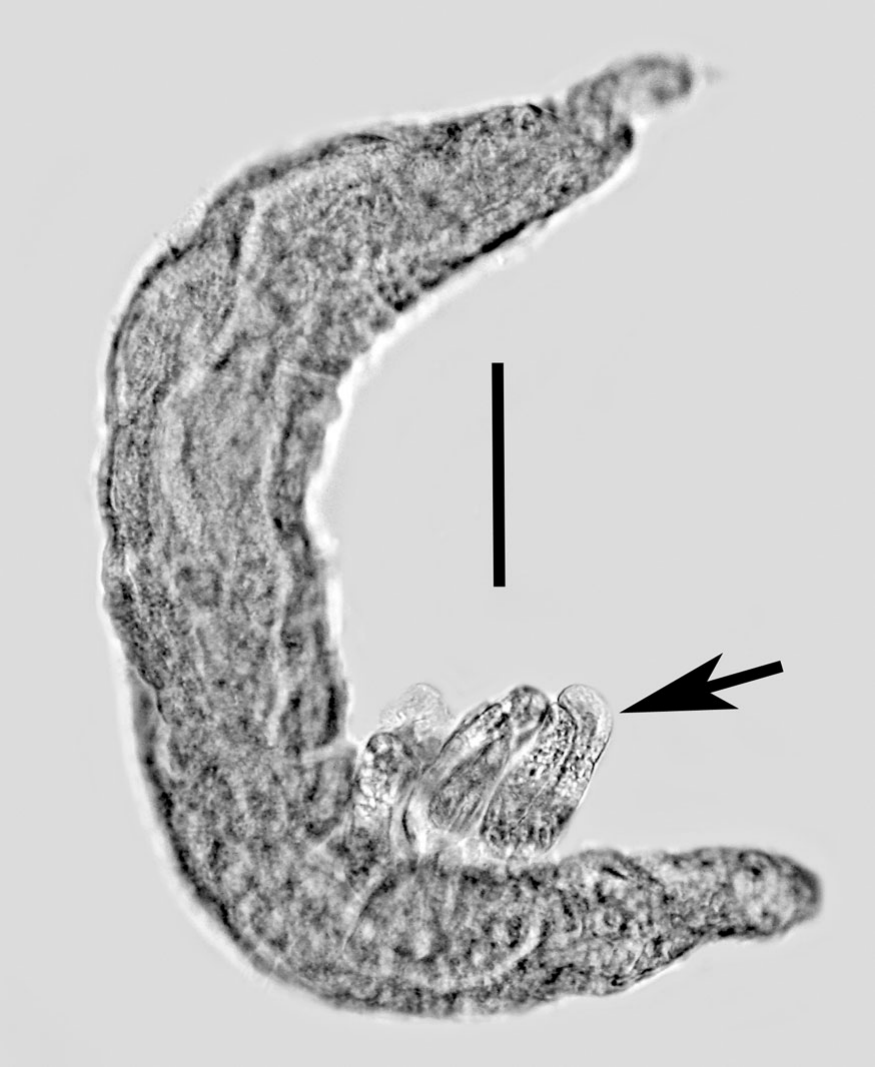
*Tripius gyraloura* n. sp. Mature parasitic female that has deposited three eggs (arrow). *Scale-bar*: 21 µm

The variability in their body size and shape in the parasitic female of *Tripius gyraloura* is quite striking. The body can be quite narrow and elongate (Figs. 5, 6) as well as robust (Fig. 7). It is likely that body shape is dependent on the amount of food available when the nematode first enters the haemocoel of its host and initiates growth. If only one or several infective stages enter, there is probably a sudden growth in length, whereas if several nematodes enter at approximately the same time, body size is reduced.

Protrusion of uterine cells through the vulva occurs in several other nematode species besides members of the genus *Tripius* such as the bumblebee parasite, *Sphaerularia bombi* (Dufour, 1837) (see Leuckart, 1887; Poinar & van der Laan, 1972) and members of the tylenchid genus *Sphaerulariopsis* Wachek, 1955 that parasitise bark beetles (Coleoptera: Scolytidae) and anobiid beetles (Coleoptera: Anobiidae) (Poinar, 1975).
